# 3-D geometric imaging of the trunk in normal adolescents and age-matched patients impaired with idiopathic scoliosis: selected effects of conservative intervention according to Schroth

**DOI:** 10.1186/1748-7161-8-S1-O20

**Published:** 2013-06-03

**Authors:** S Stoler, H Baransi, U Givon, Z Dvir

**Affiliations:** 1 The Institute of Motor Functions, Sheba Medical Center, Ramat-Gan, Israel

## Background

Research has indicated that combining the Schroth approach with corrective bracing may lead to a reduction in the number of interventions, and a deceleration in the progression of the scoliotic curve. Yet, it is still unclear whether the postural correction improves the critical factor of trunk symmetry.

## Objectives

To investigate the effectiveness of the Schroth method on trunk symmetry, in standing, sitting, and walking, using 3-D motion pick up (3-DMP) based analysis.

## Method

Twenty three adolescents, (12 healthy and 11 with AIS) participated in the study. All AIS patients had at least one curve of 20º, and were skilled in postural corrections based on Schroth principles. Trunk symmetry was assessed using the Coda Cx1 3DMP system. Markers were applied on the head, acromioclavicular joints, suprasternal notch, scapular inferior angles, pelvis, knee, hip, lateral malleoli and the heads of the 5th metatarsus. All patients were measured twice in the same day, (with a 4-hour break between sessions), in natural sitting, standing, and walking. AIS patients were measured again in those three positions, during postural correction according to Schroth. From the test-retest paradigm the stringent cut-off for a true clinical change - the smallest real difference (SRD) - was derived.

## Results

There was a significant and high correlation between the Cobb angle and scapular transverse misalignment. AIS patients showed significant correction of scapular rotation, in standing, and the location of the center of scapulae relative to the center of the pelvis. At least 36% of the patients corrected the previous, and more than 45% that of the latter, beyond the SRD. With respect to shifting of the shoulder girdle relative to the pelvis in standing and walking, 55% of the patients have exceeded the SRD value.

## Conclusions

3DMP analysis enables identification of subtle spinal relationships, which are unrecognizable using visual inspection. Using an advanced 3DMP system, this study confirms the good reproducibility of trunk marking. Moreover, it supports the effectiveness of the Schroth approach in AIS patients who are able to reduce specific trunk asymmetries, particularly those related to shoulder girdle and scapular orientation.
